# Shedding a Light on Dark Genes: A Comparative Expression Study of *PRR12* Orthologues during Zebrafish Development

**DOI:** 10.3390/genes15040492

**Published:** 2024-04-15

**Authors:** Alessia Muscò, Davide Martini, Matteo Digregorio, Vania Broccoli, Massimiliano Andreazzoli

**Affiliations:** 1 Cell and Developmental Biology Unit, University of Pisa, 56126 Pisa, Italydavide.martini@unipi.it (D.M.);; 2 Stem Cell and Neurogenesis Unit, Division of Neuroscience, San Raffaele Scientific Institute, 20132 Milan, Italy; 3 CNR Institute of Neuroscience, 20132 Milan, Italy

**Keywords:** dark genes, neurodevelopmental disorders, eye development, ASD, ADHD

## Abstract

Haploinsufficiency of the *PRR12* gene is implicated in a human neuro-ocular syndrome. Although identified as a nuclear protein highly expressed in the embryonic mouse brain, *PRR12* molecular function remains elusive. This study explores the spatio-temporal expression of zebrafish *PRR12* co-orthologs, *prr12a* and *prr12b*, as a first step to elucidate their function. In silico analysis reveals high evolutionary conservation in the DNA-interacting domains for both orthologs, with significant syntenic conservation observed for the *prr12b* locus. In situ hybridization and RT-qPCR analyses on zebrafish embryos and larvae reveal distinct expression patterns: *prr12a* is expressed early in zygotic development, mainly in the central nervous system, while *prr12b* expression initiates during gastrulation, localizing later to dopaminergic telencephalic and diencephalic cell clusters. Both transcripts are enriched in the ganglion cell and inner neural layers of the 72 hpf retina, with *prr12b* widely distributed in the ciliary marginal zone. In the adult brain, *prr12a* and *prr12b* are found in the cerebellum, amygdala and ventral telencephalon, which represent the main areas affected in autistic patients. Overall, this study suggests *PRR12*’s potential involvement in eye and brain development, laying the groundwork for further investigations into *PRR12*-related neurobehavioral disorders.

## 1. Introduction

The term “dark genes” denotes genes that, until recently, have presented challenges in assembly or alignment when utilizing conventional sequencing techniques [1]. Although these recently sequenced regions lack functional characterization, mounting evidence suggests variants in these “dark genes” are strongly associated with various diseases, including cancer, Autism Spectrum Disorder (ASD), schizophrenia, and inflammatory diseases [1,2,3]. Consequently, precision medicine research is prioritizing the identification of disease-modifying dark genes due to their potential impact on disease etiology and treatment [4,5,6]. Understanding the roles of dark genes and their associated pathways is crucial not only for precision medicine but also for advancements in cell and developmental biology. The dark gene Proline-Rich Protein 12 (*PRR12*) has been recently identified in a cohort of young age patients as the cause of a complex and heterogeneous phenotype characterized by global developmental delay and developmental abnormalities mainly affecting the brain and the eye, such as microcephaly, microphtalmia/anophtalmia and iris coloboma [7,8,9,10]. In 54% of the described cases, this clinical presentation appears in comorbidity with cognitive decline compatible with intellectual disability (ID) and a wide spectrum of behavioral alterations that range in ASD and Attention Deficit/Hyperactivity Disorder (ADHD) [7,8,9,10]. Furthermore, recent works have highlighted *PRR12* contribution in gliomas malignancy through control of cell survival and metastatic migration via the RAD21/MIR4697HG-miR7665p/PRR12 axis [11]. *PRR12* encodes for a nuclear protein particularly enriched in the brain, specifically in the cerebellum, lobes and cerebral cortex [8]. It seems to exert its yet undefined role during early development, as it was isolated in human fetal brains, and it was found highly expressed in the embryonic brain of E15 mice compared to postnatal day P1, as well as in P1 rat brains [8]. Another minor non-protein-coding *PRR12* isoform, indicated as *PRR12*-202, generated by alternative splicing, is instead predicted to be involved both in nucleus-cytoplasm cross-talk, as it was found inside and outside the nucleus, but also in cell signaling as a synaptic and perisynaptic machinery component [7,8]. Nevertheless, in the available cohort, there is no notable distinction in the clinical characteristics between patients with variants affecting the main transcript alone or both isoforms [9]. Structurally, PRR12 consists of an N-terminally located glycine-rich and several proline-rich regions, presumably useful for the formation of larger protein complexes as they can be recognized and bound by specific modules such as SH3, WW and EVH1 [7,12]. Moreover, the presence of two AT-hook DNA binding domains may serve to scaffold such protein complexes to DNA [7,8,9,12]. The presence of an N6-lysine acetylation site indicates a potential role for PRR12 in chromatin remodeling, as such modifications are frequently observed in transcriptional cofactors [8]. Similarly, its multiple phosphorylation sites are suggestive of an epigenetic regulation propensity and justify PRR12 involvement in the complex phosphoproteome during embryonic human life [8,9,13]. Not surprisingly, a high coexpression rate has been observed between *PRR12* and transcription and chromatin regulators, such as SET-domain and bromodomain-containing proteins [7,8]. Overall, *PRR12* function appears to be particularly important during early brain development and highly correlated to neuron proliferation and survival through a mechanism involving its participation in the protein interactome of the epigenetic machinery [7,8]. However, most of the above-mentioned considerations are inferences arising from sequencing and transcriptomic analysis or clinical observation of patients displaying *PRR12* variants, while a classical experimental approach to the study of this dark gene is still lacking. As zebrafish is emerging as the model of choice for studying early development, particularly in the context of the nervous system, this work aims to provide the first comprehensive spatiotemporal expression pattern of *PRR12* orthologs in this vertebrate model, thus paving the way for future investigations into the role of *PRR12* in both physiological and pathological conditions.

## 2. Materials and Methods

### 2.1. Zebrafish Husbandry

All the experiments were performed on wild-type zebrafish AB strain embryos and 10–12-month-old adults, in accordance with the standard rules defined by the Local Commitment for Animal Health (authorization n. 99/2012-A, 19 April 2012) and following the Italian and European rules on animal care (EU directive 2010/63/EU). Fish were kept in 3 L tanks with 28 °C warm water in a circulating system maintained at pH 7.0–7.5 and conductivity in a range of 450–550 µS. Adult animals were maintained under a 14/10 h light/dark cycle and fed twice per day with a combination of granular food and freshly prepared *Artemia* sp. Breeding of adult zebrafish was carried out by natural crosses. Freshly spawned embryos were collected in Petri dishes containing E3 medium (NaCl 5 mM, KCl 0.17 mM, CaCl_2_ 0.33 mM, MgSO_4_ 0.35 mM; pH 7.2; 0.2 mg/L methylene blue in deionized water) until the desired developmental stage was reached. Embryo staging was performed according to Kimmel et al. [14].

### 2.2. Bioinformatic Analysis

The sequence of the genes encoding for *PRR12* isoforms in humans and zebrafish (*prr12*) was retrieved from the Zebrafish Information Network (ZFIN) [15] and Ensembl (release 108, accessed on December 2022) within the Human (Genome Assembly: GRCh38.p13) and *Danio rerio* Genome Browsers (Genome Assembly: GRCz11). For in silico analysis, the accession numbers reported in the following table were used:

**Gene Annotation****Transcript Annotation***PRR12*-201ENSG00000126464ENST00000418929.7*PRR12*-202ENSG00000126464ENST00000593853.1*prr12a*-201ZDB-GENE-041111-223; ENSDARG00000074229ENSDART00000110514.6*prr12a*-202ENSDARG00000074229ENSDART00000152396.2*prr12b*-201ZDB-GENE-130625-2; ENSDARG00000075849ENSDART00000156464.3*prr12b*-202ENSDARG00000075849ENSDART00000188447.1

Nucleotide sequences were compared to the non-redundant sequences present at the National Center for Biotechnology Information (NCBI) using the Basic Local Alignment Search Tool (BLAST) [16,17]. 

Schematic representations of genes were created with a freely available Exon-Intron Graphic Maker online tool [16]. 

Multiple sequence alignments of proteins and protein domains were performed using the Align Clustal Omega-based tool within the UniProt database [18,19]. The following UniProt IDs were used: human PRR12 (Q9ULL5), zebrafish Prr12a-201 (A0A0R4IRJ4), Prr12b-201 (X1WDQ3), and Prr12b-202 (A0A2R8PZ98).

To find evidence for the conservation of synteny, a comparison of genes neighboring human *PRR12* and zebrafish chromosome 3 and 12, the Genomicus database [20] (DYOGEN group, version 100.01, accessed on September 2023) was used. The phylogenetic tree showing *qser1* conservation among humans and zebrafish was retrieved from Ensembl (release 111, accessed on April 2024).

RNA-Seq data for *prr12a* and *prr12b* expression profiling in embryonic and larval stages were retrieved from White et al. [21] within the Expression Atlas database [22].

### 2.3. Adult Brain and Eyes Dissection

Adult zebrafish (10–12 months of age) were euthanized through anesthetic (MS222, Sigma-Aldrich, St. Louis, MO, USA) overdose. The head was isolated by cutting with a sterile scalpel at the level of the anterior fins. Soft tissues were removed from the ventral side of the skull with surgical forceps. The skull was then opened, and the brain (1 brain/sample) and eyes (2 eyes from the same individual/sample) were transferred into TRIzol^®^ reagent (Invitrogen, Waltham, MA, USA) for RNA extraction or fixed for 6 h in 4% paraformaldehyde (PFA, UN22B) at 4 °C for in situ hybridization (ISH) experiments. 

### 2.4. RNA Extraction, RT-PCR and RT-qPCR

Total RNA extraction was performed with the guanidine isothyocianate/phenol method. Frozen pooled embryos (30 embryos/sample), dissected brain and eyes, were lysated in TRIzol^®^ reagent.

Total RNA was extracted and purified using RNAeasy Plus Mini (Qiagen, Hilden, Germany) according to the manufacturer’s instructions. RNA concentration and purity were determined using DeNovix™ (Wilmington, DE, USA) spectrophotometer. One microgram of RNA was retrotranscribed using the QuantiTect Reverse Transcription Kit (205311, Qiagen). cDNA was used for both RT-PCR and RT-qPCR amplification. Primers were designed using the Primer-BLAST tool [23] in the NCBI browser. Primer pair sequences specific to each splice variant are listed as follows: 

*prr12a*-201: FWD (5′-GAGAGTGGAGGCGAAGGCAT-3′); 

REV (5′-GGCTCTCGTCCAGTCCGAAG-3′)

*prr12a*-202: FWD (5′–GCATGCAGTGCTAATGTGAGTGTG–3′);

REV (5′-AGCATCTCCACATAGCTGCGG-3′)

*prr12b*-201; FWD (5′-AGATGTCCTGGGAGGGAGACA-3′); 

REV (5′-CGTGGACGTGCACCCATCTA-3′)

*prr12b*-202: FWD (5′-ATGTCCTTCAGGGAGGATCAAATG-3′);

REV (5′-ATGTCCTTCAGGGAGGATCAAATG-3′)

*rps11*: FWD (5′-ACAGAAATGCCCCTTCACTG-3′); 

REV (5′-GCCTCTTCTCAAAACGGTTG-3′)

Gene expression levels were evaluated using the QuantStudio™ 3 System (Applied Biosystems by Thermo Fisher Scientific, Waltham, MA, USA) and SYBR Green method (SensiMix™ SYBR^®^ NO-Rox Kit; Meridian Bioscience, Cincinnati, OH, USA), according to manufacturer’s protocol. Real time PCR was conducted with the following thermal cycle: 95 °C 10 min initial denaturation, followed by 40 cycles of 95 °C 15 s, 60 °C 15 s, 72 °C 15 s. Relative expression of *prr12a*-201, *prr12a*-202 and *prr12b*-201 mRNAs was calculated with the ∆Ct method using *rps11* as reference gene.

### 2.5. prr12a and prr12b Clones and Whole-Mount In Situ Hybridization (WISH)

To clone cDNA fragments specific for *prr12a-201* and *prr12b-201*, cDNA from 48-hpf stage embryos was used in PCR amplifications with the following primers:

*prr12a*-201: FWD (5′-ACACCAACCCTCACATTCCC-3′);

REV (5′-TTCGGGAACACACCCTTACG-3′)

*prr12b*-201: FWD (5′-TCAGCCCTGAACCACCAATG-3′);

REV (5′-GTCCGAAGGGCCATCTTCAA-3′)

The amplification conditions were the following: 3min at 95 °C, 30 cycles at 95 °C for 30 s, 58 °C for 30 s, 72 °C for 1.5 min, followed by a final extension at 72 °C for 10 min. PCR amplicons were purified using the NucleoSpin Gel and PCR Cleanup kit (Macherey-Nagel, Düren, Germany) according to the manufacturer’s protocols and subjected to ligation in the pGEM-T vector system (Promega, Fitchburg, WI, USA) following the producer’s manual. Ligation products were used to transform *Escherichia coli* DH5α-competent cells following standardized laboratory procedures. Cloned bacterial plasmid DNA was recovered using the NucleoBond Xtra Midi kit (Macherey-Nagel, Düren, Germany) and verified for the sequence and orientation of each insert. 

Antisense and sense RNA probes were obtained by in vitro transcription of the cloned *prr12a*-201 and *prr12b*-201 cDNAs with either T7 or SP6 RNA polymerase using the DIG RNA labeling Mix kit (Roche, Basilea, Switzerland).

WISH was performed according to a previously described protocol [24], with some modifications. Briefly, twenty-five embryos at the desired developmental stage were fixed for 1 h at room temperature (RT) in 4% PFA and dehydrated through ascending (25–50–75%) Phosphate Buffer Saline (PBS)-0.5% Triton X-100 (Sigma-Aldrich)/methanol (Carlo Erba, Milan, Italy) solutions and stored in 100% methanol at −20 °C. The day of the experiment, samples were rehydrated and permeabilized with 10 µg/mL proteinase K (P2308, Roche). Embryos were then incubated overnight (O/N) at 68 °C in hybridization buffer containing 300 ng/mL DIG-labeled probes. The day after, samples were washed with Hybe Wash (50% deionized formamide and 1X Sodium Citrate buffer, SSC) and SSC, then incubated with anti-DIG antibody conjugated with alkaline phosphatase (1:2500, 11093274910, Roche) for 4 h at RT in a blocking solution made of 2% Blocking Reagent (Roche) in Maleic Acid Buffer 1X (MAB1X, 100 mM maleic acid + 150 mM NaOH in deionized water, pH 7.5). Staining was performed through sample incubation in BM Purple solution (Roche), kept in the dark at RT until signal development. Hybridized embryos were fixed in 4% PFA and stored in 100% ethanol at −20 °C. WISH images were taken using a Nikon SMZ1500 stereomicroscope and processed using the NIS-Elements Viewer 5.21 software (Nikon Instruments, Tokyo, Japan).

### 2.6. ISH on Adult Brain Cryosections

To obtain frozen sections, fixed brains were cryoprotected in 30% sucrose in PBS and embedded into Killik O.C.T. (Bio Optica, Milan, Italy) and then placed at −80 °C for quick freezing. Brains were sectioned into 12 µm-thick slices using the HM525 NX cryostat (Epredia, Portsmouth, NH, USA) and finally collected onto polarized SuperFrost™ Plus Adhesion Microscope Slides (Epredia).

ISH on frozen tissue sections was performed as described in Gabellini et al. [25], with some modifications. Cryosections were thawed and washed in PBT (PBS + 0.5% Triton X-100) and incubated with either 300 ng/mL *prr21a*-201 or *prr12b*-201 antisense probes at 65 °C O/N. Slides were then rinsed at 65 °C in Hybe Wash and MAB + 0.1% Tween20 (Sigma-Aldrich) (MABT) solutions at RT. After 1 h-long equilibration at RT in the previously described blocking solution added with 20% lamb serum, slides were incubated with anti-DIG antibody (diluted 1:2500 in the blocking solution) at 4 °C O/N. Slides were stained in BM Purple Solution in the dark at RT. After the staining procedure, images were acquired using a Nikon Eclipse Ti microscope (Nikon Instruments).

### 2.7. Statistical Analysis

The data are reported as the sum or as a representative of at least two independent experiments. Statistically significant differences in gene expression analysis were calculated either by one-way ANOVA or *t* test, followed by the appropriated post hoc analysis. Significance was established at *p* < 0.05. All graphs were prepared using GraphPad PRISM Software version 6.0 (Boston, MA, USA).

## 3. Results

### 3.1. In Silico Analysis of Zebrafish PRR12 Orthologs

**Figure 1:**
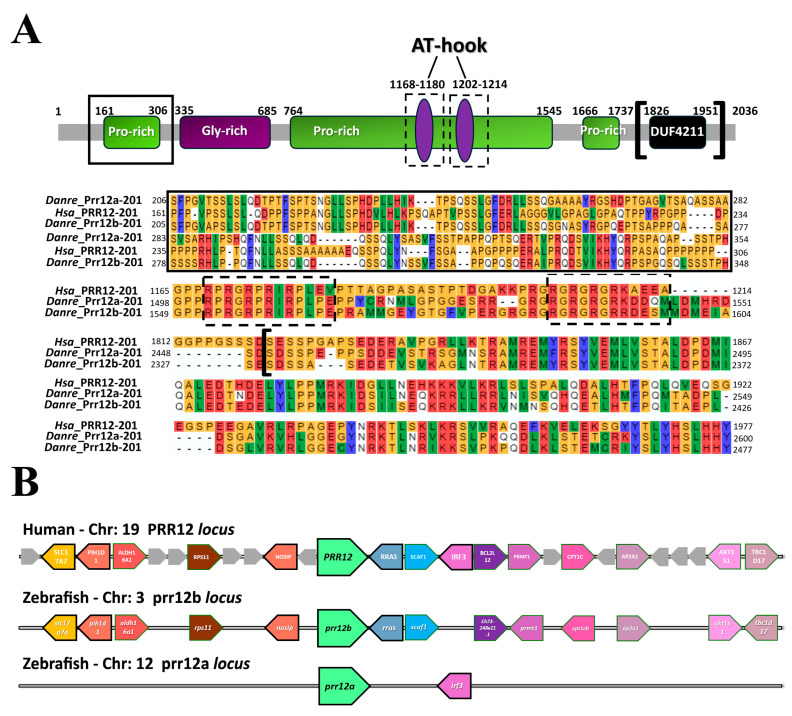
**Multiple in silico approaches unveil conservative features between *PRR12* and its orthologs in zebrafish.** (**A**) Schematic representation of human PRR12 protein highlighting the main acknowledged domains (modified from Chowdhury et al. [7]), together with a multiple sequence alignment of the amino-terminal proline-rich (black box; 35% identity Prr12a-201 and Prr12b-201 vs. PRR12), AT-hook (black dashed-line boxes; AT-hook 1168–1180aa: 91.66% identity Prr12a-201 and Prr12b-201 vs. PRR12; AT-hook 1202–1214: 66.67% identity Prr12a-201 and Prr12b-201 vs. PRR12) and DUF4211 (black squared brackets; 60% identity Prr12a-201 and Prr12b-201 vs. PRR12) functional motifs of the human and zebrafish Prr12a-201 and Prr12b-201 proteins. Abbreviations: ***Hsa*:** *Homo sapiens*; ***Danre*: *Danio rerio***. (**B**) Syntenic relationship between human *PRR12* and zebrafish *prr12a* and *prr12b loci*. A total of six genes is conserved in *prr12b* surroundings compared to chromosome 19. A poor synteny in conversely evidenced for *prr12a* locus. **Chr:** chromosome.

The NCBI Gene database reports the presence of two zebrafish co-orthologs of the human *PRR12* gene, namely *prr12a* and *prr12b*, located on chromosome 12 and on chromosome 3, respectively. For each of the two *D. rerio prr12* genes, two splice variants are reported (Supplementary Figure S1). The *prr12a*-201 variant is organized in 15 exons and encodes for a 2532 aa protein. Conversely, *prr12a*-202 is a five-exon, non-coding, minor transcript. For the co-ortholog *prr12b*, both transcript variants, namely *prr12b*-201 and *prr12b*-202, are protein-coding. Specifically, the sixteen exons of *prr12b*-201 encode for a 2656 aa product, while the eight exons of *prr12b*-202 encode for a shorter, 248 aa peptide (Supplementary Figure S1). Aiming at unravelling the subsistence of any functional conservation among the human protein and its zebrafish co-orthologs, aminoacidic sequence alignment was performed in order to analyze the rate of conservation of the PRR12 domains denoted in Chowdhury et al. [7]. Overall, an alignment of the deduced protein sequences shows that, compared to human PRR12, Prr12a-201, Prr12b-201 and Prr12b-202, share an identity of 43.07%, 58.15% and 50.08%, respectively. Amino-terminal proline (Figure 1A) and glycine-rich (Supplementary Figure S2A) sequences for both 201 zebrafish proteins shared around 32–34% identical residues compared to the human counterpart, while a mere 26–31% identity was found for the carboxy-terminal proline-rich region (Supplementary Figure S2C). A slight increase at 44–45% was seen instead for the intermediate proline-rich domain (Supplementary Figure S2B). Interestingly, the 1826–1951 aa region hosting the DUF4211 domain appeared highly conserved among all the zebrafish Prr12 proteins, reaching a ~60% identical composition in all isoforms (Figure 1A). On the same line, the 1168–1180aa and the 1202–1214aa AT-hook DNA binding domains shared 91.66% and 66.67% identity, respectively, for both 201 isoforms compared to the human protein (Figure 1A). Previous works have highlighted the importance of specific aminoacidic residues representing putative targets for post-translational modifications [8,9]. Lysine residue 402 was found to be conserved in Prr12a-201 and Prr12b-201 (Supplementary Figure S2D). Conversely, serine 1104 (1925 aa site in the more recent release) did not have a correspondent in any of the zebrafish proteins (Supplementary Figure S2E). The analysis of the chromosomal regions surrounding the human and zebrafish genes showed a conserved synteny between the human chromosome 19 region harboring the *PRR12* gene and the *D. rerio* chromosome 3 region, where the *prr12b* ortholog is located (Figure 1B). Indeed, a total of six genes in the human *PRR12* locus have a corresponding ortholog in their *prr12b* surroundings. Concerning the *prr12a* locus, located in chromosome 12, only one gene, *irf3*, is maintained compared to the *PRR12* region. Interestingly, signaling pathways implying *irf3* maternal activation during development have been associated with the onset of neurodevelopmental disorders (NDDs) [26,27]. The epigenetic regulator *QSER1* is reported in the literature as an essential paralog of *PRR12* in the mammalian genome [28]. This relationship between *qser1* and *prr12* is also preserved in zebrafish (Supplementary Figure S3). 

### 3.2. Temporal Expression: prr12a and prr12b Activation Is Differentially Regulated during Zebrafish Development

**Figure 2:**
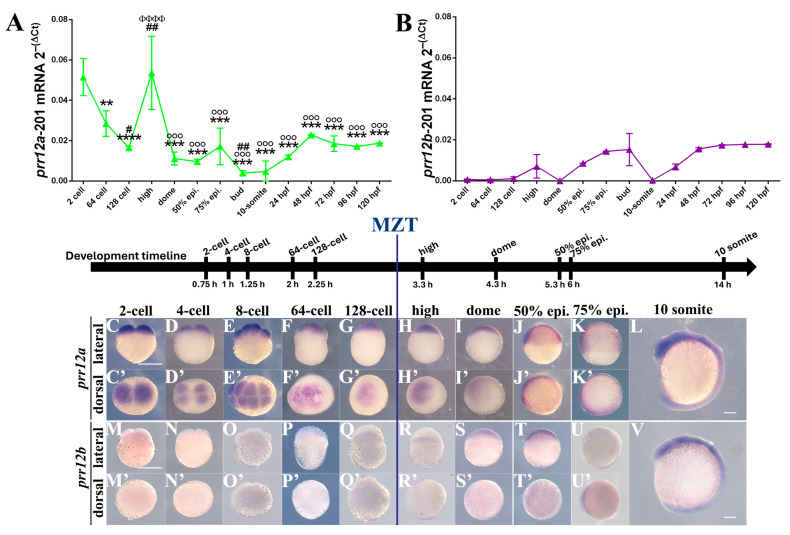
***prr12a* and *prr12b* display a different spatio-temporal expression pattern during zebrafish early development.** (**A**,**B**) Real-time PCR profile of *prr12a-*201 (**A**) and *prr12b-*201 (**B**) at increasing developmental stages (epi.: epiboly; hpf: hours post fertilization). Ct values for each timepoint are normalized to *rps11* gene. Asterisks (*) indicate statistical comparisons between each stage and 2 cell stage; hash marks (#) indicate statistical comparisons between each stage and 64 cell stage; Phi (Φ) indicate statistical comparison between each stage and 128 cell stage; degree symbol (°) indicate statistical comparison between each stage and high stage. Data are expressed as mean ± SD. ANOVA followed by Tukey post hoc test: ** *p* < 0.01; *** *p* < 0.001; **** *p* < 0.0001; # *p* < 0.05; ## *p* < 0.01; ΦΦΦΦ *p* < 0.0001; °°° *p* < 0.0001. *n* = 2 independent experiments. (**C**–**V**) WISH using *prr12a*-201 anti-sense (**C**–**L**) and *prr12b*-201 (**M**–**V**) anti-sense probes at early developmental stages. Lateral (**C**–**L**,**M**–**V**) and dorsal (**C’**–**K’**,**M’**–**U’**) views of the hybridized embryos. Scale bars: 250 µm. Abbreviations: epi.: epiboly h: hours; MZT: maternal-to-zygotic transition.

To analyze the temporal expression pattern of *prr12a* and *prr12b* during embryonic development, we performed a real-time reverse transcription-polymerase chain reaction (RT-qPCR) assay on cDNAs obtained from zebrafish embryos at different developmental stages. The transcript for *prr12a*-201 is detected as early as the two-cell stage, indicating that this isoform is maternally provided. After this peak, transcript abundance decreases steeply during the subsequent zygotic divisions and rises again at the high stage, thus returning to its maximum level of expression just after the onset of the maternal-to-zygotic transition (MZT). Subsequently, from gastrulation stages onwards, *prr12a*-201 results are weakly expressed and fluctuating (Figure 2A). Conversely, the *prr12a*-202 isoform is generally poorly expressed during zebrafish development (Supplementary Figure S5A). Moreover, in contrast to *prr12a*-201 early onset, the *prr12a*-202 transcript starts to be detected in late gastrulation, peaks at the bud stage, but then sharply decreases and remains steady during both pre- and post-hatching phases. On the other hand, *prr12b*-201 displays a low expression level with no significant changes throughout the embryonic and larval stages (Figure 2B), whereas *prr12b*-202 could not be detected in any of the considered developmental stages. Overall, our RT-qPCR data are consistent with, and extend, the RNAseq results for zebrafish *prr12* isoforms available from the Wellcome Trust Sanger Institute [21,22] (Supplementary Figure S4).

### 3.3. Spatial Expression: prr12a and prr12b Display Distinct Expression Patterns in the Developing and Adult Nervous System

To determine the spatial expression of zebrafish *prr12a* and *prr12b*, we performed ISH using probes specific for each of the most represented isoforms, namely *prr12a*-201 and *prr12b*-201, hereafter referred to in this paragraph as *prr12a* and *prr12b*, respectively. Spatial expression comparisons for the two genes will be discussed considering four defined developmental timeslots spanning embryonic and larval stages, while a separate subsection will be dedicated to pattern description in the adult brain.

#### 3.3.1. Zygote (0.0 hpf)–MZT (3.00 hpf)

Prior the activation of the zygotic genome, maternally derived *prr12a* can be detected in two-cell stage embryos as a diffuse signal covering both cells (Figure 2C,C’). A homogeneously distributed expression pattern characterizes the two subsequent blastomere divisions at four- and eight-cell stages (Figure 2D,E,D’,E’). In 64-cell embryos, the *prr12a* transcript is mainly restricted to the apical part of the animal pole (Figure 2F,F’), becoming again more diffuse in the rising blastodisc at 128-cells (Figure 2G,G’). No signal was detectable for *prr12a* sense-probe, used as an experimental control at the same developmental stages (Supplementary Figure S6A). On the contrary, *prr12b* transcript results were undetectable in the same initial phases of cleavage (Figure 2M–Q).

#### 3.3.2. Mid-Blastula (3.33 hpf)–Segmentation (14 hpf)

In the mature blastodisc, *prr12a* transcripts are uniformly distributed in the animal pole at high and dome stages (Figure 2G,H), and persist during gastrula development, labelling the enveloping layer in 50%- and 75%-epiboly embryos (Figure 2J,K). The beginning of the epiboly movements represents the timepoint at which the prr12b transcript becomes observable as a subtle signal in the domed deep cell layer (Figure 2S,S’). A more noticeable expression characterizes 50%-epiboly embryos, in which prr12b is distributed along the thick blastoderm (Figure 2T,T’). Nonetheless, *prr12b* activation still appears unsteady in the gastrulation phase, as the signal is partially lost at 75% epiboly (Figure 2U,U’). With the onset of segmentation, *prr12* tissue specificity becomes more evident for both isoforms. The *prr12a* and *prr12b* probes clearly label the brain primordium in the anteriormost part of the embryo, while the signal vanishes, moving caudally along the trunk (Figure 2L,V). Control sense probes for *prr12a* and *prr12b* resulted in the absence of a signal at each of the considered timepoints (Supplementary Figure S6A,B). 

#### 3.3.3. Pharyngula (24 hpf)

**Figure 3:**
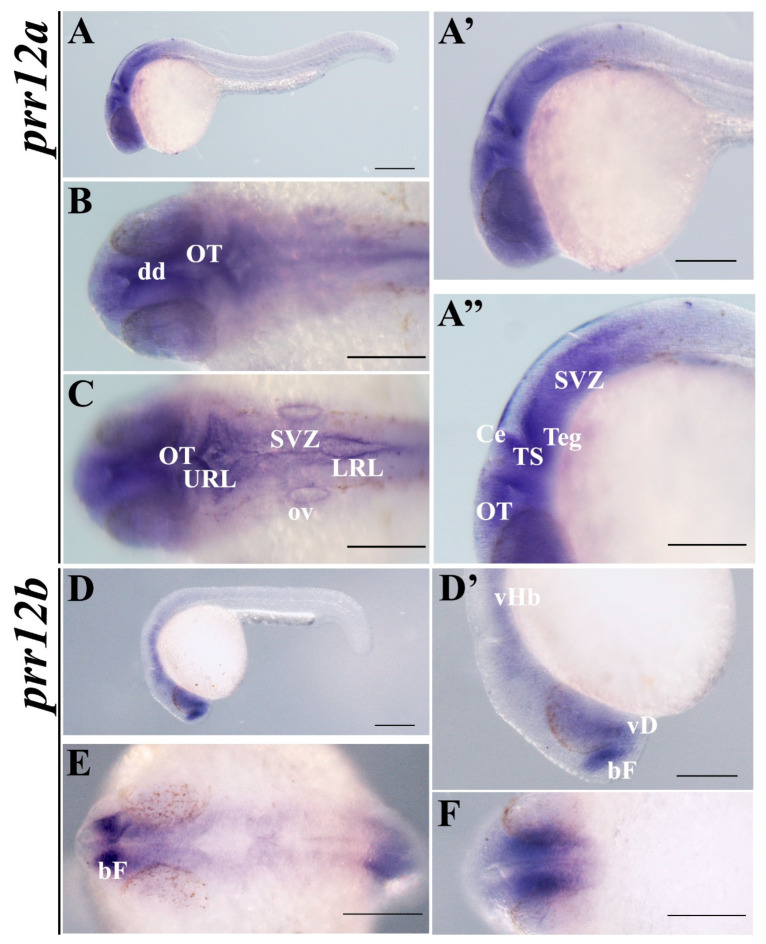
***prr12a-201* and *prr12b-201* are distributed differently in 24 hpf embryos.** WISH analysis of *prr12* expression at 24 hpf stage. (**A**,**D**) Lateral views of 24 hpf hybridized embryos and magnification of heads shown in (**A’**) and (**D’**). (**A’’**) Enlargement of *prr12a*-201-positive periventricular area. (**B**,**C**) forebrain and hindbrain dorsal views of *prr12a* hybridized embryos. (**E**,**F**) Dorsal and ventral views, respectively, of *prr12b*-201 hybridized embryos. Scale bars: 500 µm. Abbreviations: **bF:** basal forebrain; **Ce:** cerebellum; **dd:** dorsal diencephalon; **LRL:** lower rhombic lip; **OT:** optic tectum; **ov:** otic vesicle; **SVZ:** subventricular zone; **TS:** torus semicircularis; **Teg:** tegmentum; **URL:** upper rhombic lip; **vHb:** ventral hindbrain; **vD:** ventral diencephalon.

At 24 hpf, the expression pattern of the two *prr12* paralogues diverges consistently. In particular, while *prr12a* expression covers most of the central nervous system (CNS), excluding the posterior trunk and tail (Figure 3A,A’), *prr12b* expression is restricted to three main districts, corresponding to the basal forebrain, the ventral diencephalon and the ventral hindbrain (Figure 3D,D’). Notably, no signal for *prr12b* was detected in the developing retina (Figure 3D’,E,F), which instead appears to express *prr12a* (Figure 3A’,B,C). At this stage, *prr12a* is clearly detectable in the three major divisions of the embryonic brain, partially overlapping with *prr12b*. Undoubtedly, the main distinguishing features between the two orthologs reside in the presence of the *prr12a* transcript in the optic tectum, in the ventrally located tegmentum in the midbrain, and in the interconnecting torus semicircularis (Figure 3A’’). Moreover, in the hindbrain, *prr12a* mRNA appears enriched in the cerebellar primordium and, particularly, in the subventricular and upper and lower rhombic lip germinal zones in close association with the IV ventricle (Figure 3B,C). Finally, a fine signal can be detected in the tissue surrounding the optic vesicle (Figure 3C).

#### 3.3.4. Hatching (48 hpf–72 hpf)

**Figure 4:**
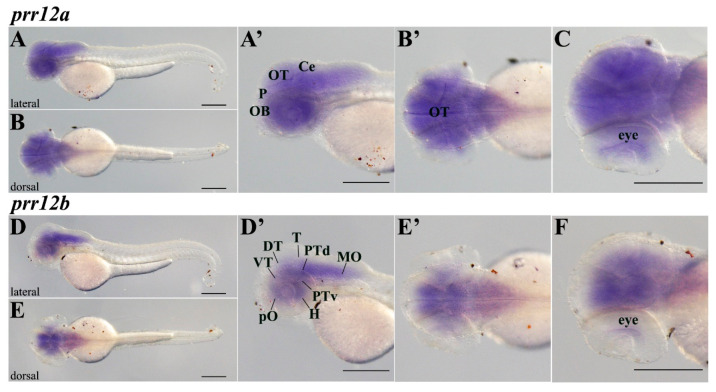
***prr12* co-orthologs cover distinct domains of 48 hpf central nervous system.** WISH evaluation of *prr12* expression at 48 hpf. Scale bars: 500 µm. (**A**,**D**) lateral and (**B**,**E**) dorsal views of hybridized embryos. (**A’**,**D’**,**B’**,**E’**) represent the enlargements of the heads of the embryos shown in (**A**,**D**,**B**,**E**), respectively. (**C**,**F**) are the same embryos shown in (**B’**,**E’**), respectively, slightly tilted on a side and analyzed at higher magnifications to better show the hybridization signal in the eye. Abbreviations: **DT:** dorsal thalamus; **H:** hypothalamus; **MO:** medulla oblongata; **P:** pallium; **PTd:** dorsal part of posterior tuberculum; **PTv:** ventral part of posterior tuberculum; **T:** mesencephalic tegmentum; **VT:** ventral thalamus.

The secondary neurogenesis wave determines an enlargement of *prr12b* midbrain domains in 48-hpf embryos. Indeed, while the telencephalic signal is constrained to the preoptic region, the diencephalic derived dorsal and ventral thalamus and posterior tuberculum are well represented, and the signal develops along the dorso-ventral axis, extending from the ventrally located hypothalamus to the dorsalmost pretectal area. Posteriorly, *prr12b* localization in the hindbrain remains confined to basal proliferative territories, as in 24-hpf embryos (Figure 4D,D’). The distribution of *prr12a* transcript in the telencephalon and diencephalon seems to partially recapitulate *prr12b* expression at 48 hpf, although, as a point of divergence, it also spreads anteriorly, in the pallium and olfactory bulb, and dorsally, reaching the optic tectum and the cerebellar plate (Figure 4A,A’). Similarly, in the hindbrain, *prr12a* mRNA covers both basal and apical areas. Notably, both isoforms are expressed in the eye at this stage, although *prr12b* to a lesser extent, apparently in the most internal layers of the neural retina (Figure 4B’,C,E’,F). 

**Figure 5:**
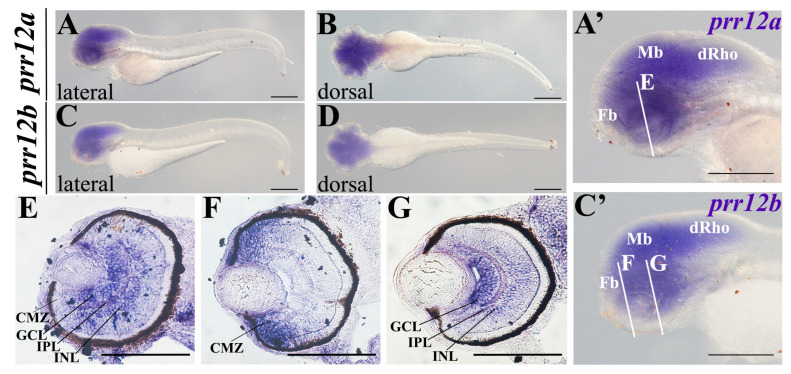
***prr12a-201* and *prr12b-201* territories converge in 72-hpf embryos.** WISH (**A**–**D**,**A’**,**C’**) and ISH on cryosectioned larval heads (**E**–**G**). (**A**,**C**) lateral and (**B**,**D**) dorsal views of hybridized larvae. (**A’**,**C’**) enlargements of the heads of the embryos, respectively shown in (**A**,**C**). White lines indicate the section planes shown in (**E**–**G**). Scale bars in (**A**–**D**) and (**A’**–**C’**): 500 µm; Scale bars in (**E**): 250 µm. Abbreviations: **CMZ**: ciliary marginal zone; **dRho**: dorsal rhombencephalon; **Fb**: forebrain; **GCL**: ganglion cell layer; **INL**: inner nuclear layer; **IPL**: inner plexiform layer; **Mb**: midbrain.

At 72 hpf, *prr12a* and *prr12b* expression patterns overlay, becoming highly similar, as the *prr12b* transcript is now detectable in the midbrain and dorsal rhombencephalon, in the forebrain and in the neural retina (Figure 5A–C’). As *PRR12* variants have been associated with visual system developmental defects involving coloboma, microphtalmia and anophtalmia [7,9,10], we decided to conduct an in-depth analysis of *prr12* distribution in the embryonic eye at 72 hpf, when retinal lamination is completed and the retina is functional (Figure 5E–G). Both transcripts are particularly enriched in the retinal ganglion cell layer (GCL) and, to a lesser extent, in the inner region of the inner nuclear layer (INL), as well as in the intermediate synaptic area of the inner plexiform layer (IPL) (Figure 5E–G). Interestingly, *prr12b* (Figure 5F,G) but not *prr12a* (Figure 5E) is found widespread in the retinal stem cell niche of the ciliary marginal zone (CMZ), which hosts proliferating retinoblasts (Figure 5F). 

#### 3.3.5. Adult Brain

**Figure 6:**
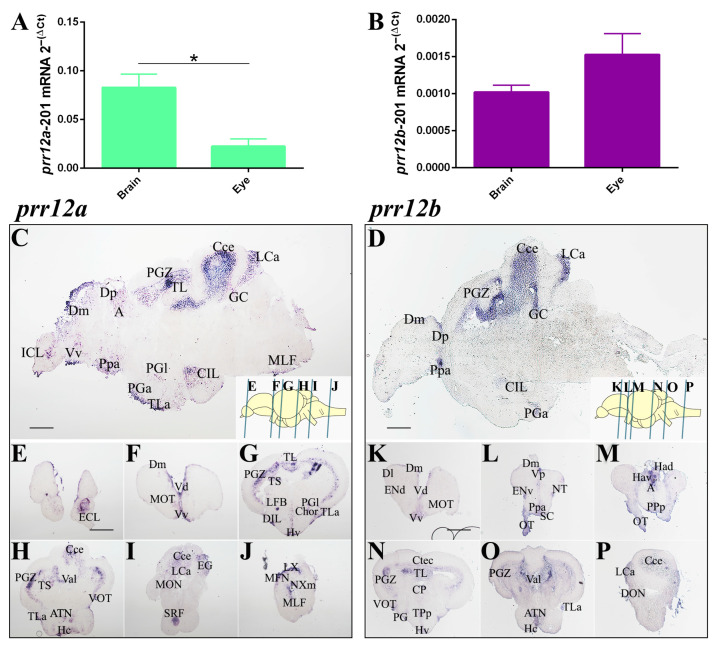
***PRR12* zebrafish orthologs are expressed in adult brain and eye.** (**A**,**B**) Relative quantification of *prr12a-201* (**A**) and *prr12b-201* (**B**) by RT-qPCR. Ct values are normalized to *rps11* gene. Data are presented as mean ± SEM. Unpaired *t* test. * *p* < 0.05. *n* = 3 independent experiments. (**C**–**P**) ISH on sagittal (**C**,**D**) and transversal (**E**–**P**) adult brain cryosections. *prr12a-201* and *prr12b-201* mRNAs are found in proliferative niches and in nuclei variably associated with the olfactory, attentive, emotional and visual systems. Blue bars in the schematic depictions of the sagittal brain shown on the bottom right in (**C**,**D**) pictures, indicate the cutting planes of the cryosections shown in (**E**–**P**). In (**C**,**D**), as well in the relative brain schemes, anterior is to the left and dorsal is to the top. Scale bars: 50 µm. Abbreviations: **A**: anterior thalamic nucleus; **ATN**: anterior tuberal nucleus; **Cce**: cerebellar corpus; **CG**: central gray; **Chor**: horizontal commissure; **CIL/DIL**: central/dorsal nucleus of the inferior lobe; **Ctec**: tectal commissure**; CP:** central posterior nucleus of dorsal hypothalamus; **Dm/l/p**: medial/lateral/posterior zone of dorsal telencephalic area; **ECL/ICL**: external/internal cellular layer of olfactory bulb; **ENv**: entopeduncular nucleus—ventral part; **Hav/Had**: ventral/dorsal habenular nucleus; **Hc/d**: caudal/dorsal zone of periventricular hypothalamus; **Lca**: caudal lobe of cerecellum; **LFB**: lateral olfactory tract; **LX**: vagal lobe; **MFN**: medial funicular nucleus; **MLF**: medial longitudinal fascicle; **MOT:** medial olfactory tract; **N**X**M**: vagal motor nucleus; **PGZ**: periventricular gray zone of optic tectum; **TL**: longitudinal torus; **PGa/l**: anterior/lateral preglomerular nucleus; **Ppa/p/v**: parvocellular preoptic nucleus-anterior/posterior/ventral part; **SRF**: superior reticular formation; **TLa**: lateral torus; **TPp**: periventricular nucleus of posterior tuberculum; **TS**: semicircular torus; **Val**: lateral division of valvula cerebelli; **VOT/OT**: ventrolateral/optic tract; **Vv**: ventral nucleus of ventral telencephalic area.

RT-qPCR profiling indicates that *PRR12* zebrafish orthologs are also expressed in the brain and eyes of adult individuals. In particular, *prr12a*-201 (Figure 6A) and *prr12a*-202 (Supplementary Figure S5B) isoforms exhibit higher expression in the brain, while no significant difference between brain and eye tissues is observed for *prr12a*-201 (Figure 6B). To deeply investigate *prr12* expression in the adult brain, we performed ISH on serial sagittal (Figure 6C,D) and transversal (Figure 6E–P) cryosections. Consistent with the findings in 72-hpf embryos, both *prr12a* and *prr12b* maintain a highly similar spatial distribution within the mature CNS, primarily encompassing pallial and subpallial territories. As observed during embryonic development, both transcripts closely associate with several known proliferative niches in the adult brain. These include the tectal periventricular grey zone (PGZ), the ventral part of the ventral telencephalon (Vv) and the dorsally located posterior pallium (Dp), which corresponds to the mammalian SVZ and hippocampal subgranular zone (SGZ), respectively [29] (Figure 6C,D,F,K,N). The same consideration can be extended to the several nuclei in the ventral diencephalon, where both *prr12a* and *prr12b* are expressed. These nuclei, including the anterior tuberal nucleus (ATN), periventricular nucleus of the posterior tuberculum (TPp), and the torus longitudinalis (TL), along with the Sox2+ anterior part of the parvocellular preoptic nucleus (Ppa), have recently been discovered to possess regenerative potential [30,31] (Figure 6C,H,L,N). Another unchanged aspect compared to developmental expression pattern is *prr12* association with the visual system, winding among telencephalic and diencephalic nuclei in the adult brain, and also reinforced by transcripts presence in the optic (OT) and ventrolateral optic tracts (VOT) and the tectal torus longitudinalis (TL), which drives integration of visual information and spatial summation through a mechanism involving signals originated by retinal ganglion cells [32] (Figure 6G,H,L,M,N). Both mRNAs are also related to the path of olfactory signals. Indeed, inputs derived from the external cellular layer (ECL) convey to the Dp, defined as the olfactory pallium, while the medial olfactory tract (MOT) and the lateral forebrain bundle (LFB) guide output signals processed in pallial and subpallial olfactory regions comprising hypothalamus-associated and habenular nuclei [33,34] (Figure 6E,G,M). Indeed, while *prr12b* is the only orthologue expressed in the dorsal and ventral serotoninergic habenular nuclei (Had/Hav), both transcripts can be detected in the ventral zone of the periventricular hypothalamus (Hv) and in the preglomerular nucleus (PG) (Figure 6G,M,N). Not surprisingly, *prr12b* is also detected in the ventral part of the entopeduncular nucleus (ENv), the correspondent of mammalian ENT, representing the main telencephalic nucleus projecting to the habenula [35,36] (Figure 6L). However, based on its projection to the several pallial components of adult telencephalon, the preglomerural nucleus (PG) has been interpreted as a general sensory relay station, homologous to thalamic nuclei in mammals [37,38]. Embryonic *prr12* expression in tissues surrounding the midbrain-hindbrain boundary (MHB) results in transcript detection in the adult cerebellum. Indeed, *prr12a* and *prr12b* are localized in the granular layers of the corpus and valvulae cerebelli (Cce) and in the caudolateral lobe (LCa) (Figure 6C,D,H,I,P). Moreover, transcript detection in the descending octavolateral (DON) and central gray (CG) nuclei evidences *prr12* contribution to cerebellar circuitry functionality (Figure 6C,D,P).

## 4. Discussion

The dark gene *PRR12* has been recently identified as a promising candidate gene for a heterogeneous syndrome characterized by neurodevelopmental, eye and behavioral alterations mainly diagnosed in children, thus embracing some peculiar traits of ID and certain neurodevelopmental disorders, such as ASD and ADHD [7,8]. Exome sequencing data report a low tolerance for *PRR12* towards loss-of-function mutations [39]. Not surprisingly, all the variants found in the cohort of patients are heterozygous, occurring with the highest frequency in exon 4 containing a splice-acceptor site and predicted to be causative of nonsense-mediated transcript decay and protein truncation [7,9]. The aforementioned clinical features may be to some extent due to PRR12’s predicted capability to form complexes with proteins known to be involved in eye and cognitive development (SOX2, USP7) and to bridge them to DNA [7]. On the other hand, PRR12 itself may be bound and variably activated/deactivated by epigenetic regulators through acetylation and phosphorylation of key aminoacidic residues [7,8,9]. Although gene and protein sequences are curated at the primary level, the current knowledge about *PRR12* function and correlation with such a clinical picture, is scarce. To respond to the necessity of a deeper comprehension of this dark gene and to provide initial biological evidence supporting its association with the above-mentioned clinical features, we report the first description of the expression pattern of *PRR12* orthologues in the zebrafish vertebrate model. 

Overall, several pieces of evidence allow us to postulate a remarkable degree of conservation between *prr12a* and *prr12b* compared to their human counterparts. In particular, a clear synteny is observable between the human *PRR12* locus and the zebrafish *prr12b* locus in chromosome 3. It is worth mentioning the association with *nosip*, involved in eye formation and cranial cartilage development, and *rras* and *prmt1*, variably associated with CNS and eye maturation [40,41,42,43,44]. Furthermore, *prmt1* haploinsufficiency and *rps11* overexpression have been found to be related to the appearance of epileptic features in mice [45,46]. On the other hand, synteny analysis revealed that the *prr12a* locus has almost completely lost any genetic association with human chromosome 19, presumably due to the second additional round of whole genome duplication that occurred in teleosts, except for the conserved presence of the *irf3* gene. Notably, the SNP variant rs12462756 in the *PRR12* gene, which determines an increase in *PRR12* mRNA in the cerebellum, putamen and cortex, occurs in comorbidity with an *IRF3* increase in the cerebellum, and both are associated with the appearance of neuropsychiatric and cardiovascular traits and reduced cognitive performance [47]. A comparison between *prr12a*, *prr12b* and human *PRR12* shows evolutionary conservation in gene structure, including the total number and length of exons, as well as in gene sequence, notably with the highest percentage of identity borne to exon 4, which represents the most frequently targeted by mutations in the patients’ cohort. Furthermore, even in zebrafish, both *prr12a* and *prr12b* are predicted to generate two isoforms by alternative splicing, although in our hands, *prr12b-*202 was undetectable either in embryos or adult individuals. Concerning *prr12a-*202, it may resemble the human 202 isoform, as both are non-coding transcripts whose expression increases in the adult brain [7]. Thus, it is reasonable to speculate that, similarly to *PRR12-*202, it may participate in intracellular communication inside and outside the nucleus. At the protein level, the Prr12b-201 aminoacidic sequence globally presents a higher percentage (58.15%) of identical residues compared to Prr12a-201 (43.07%), with respect to the human ortholog. However, looking at the presumptive PRR12 functional domains, this difference flattens out as a null or an insignificant divergence appears from protein alignments. Specifically, the intermediate glycine- and the various proline-rich motifs are poorly (30–40% identity) conserved in Prr12 paralogs. Conversely, highly conservative are both AT-hook and DUF4211 sequences. Overall, these findings outline that Prr12a-201 and Prr12b-201 may maintain PRR12 AT-motifs recognition and DNA binding capability. In contrast, the lower concentration of repeated proline residues suggests that zebrafish Prr12 proteins may use different scaffolding strategies not involving proline-rich domain identification. Interestingly, the retention in both zebrafish proteins of the domain of unknown function DUF4211 widens the evolutionary conservation with PRR12, encompassing the *QSER1* gene. In fact, zebrafish Qser1 shares the same domain as its human ortholog and is designated as the paralog of both Prr12a and Prr12b, similar to their human counterparts.

Mammalian QSER1 is involved in the maintenance of a poised chromatin state during development, counteracting DNA hypermethylation [48]. Although no data regarding the *qser1* biological role are currently available, this evolutionary parallelism may disclose the functional implication of *prr12* in the epigenetic regulation of developmental processes, already hypothesized for the PRR12-QSER1 interaction. The preservation in both zebrafish orthologs of lysine residue 402, considered an important acetylation target and epigenetic mark, is in strong agreement with this speculated participation of *prr12* in chromatin remodeling. 

*PRR12* is highly expressed in the mammalian developing brain and decreases in post-natal life [7,8]. In zebrafish, *prr12a*-201 undoubtedly represents the dominant ortholog during the pre-gastrulation phase, being already present as a maternal transcript, a time window in which prr12b-201 is nearly undetectable. Indeed, only after the onset of the pharyngula period, *prr12a*-201 and *prr12b*-201 expression profiles become aligned. Except for some dissimilarities due to the different sensitivity of the techniques here used, the distinct temporal activation of the two zebrafish orthologs is reinforced by WISH data and corroborated by the publicly available transcriptomic profile. In contrast to the decrease in PRR12 expression reported in adult individuals, zebrafish orthologs are still considerably detected even in mature brains and eyes. This apparent divergence in the expression profiles may be explained by the largely renowned neuronal regenerative capacity that characterizes the adult zebrafish brain, a feature that is not preserved in the CNS of higher vertebrates [49,50]. This regenerative potential, presumably requires a constantly running but properly regulated self-renewal apparatus, thus reinforcing the *PRR12* linkage to cell proliferation control. Indeed, PRR12 upregulation determines an increase in cell apoptosis and a decrease in cell migration and invasion [11]. Moreover, overexpression of PRR12 and increased proliferation are also found in the lymphoblastoid cell line derived from a patient characterized by a translocation-fusion of *PRR12* and *ZMIZ1* genes [8].

However, *PRR12* association with proliferative brain zones is a subject that can undoubtedly extend to the whole zebrafish lifespan, as shown by *prr12a*-201 enrichment in the hindbrain subventricular zone (SVZ) at 24 hpf. Moreover, as previously discussed, both isoforms are vastly expressed in well-known proliferative areas in the adult brain, some of which are acknowledged as homologous to mammalian regenerative niches [29]. Notably, *prr12b-*201 seems to be associated with more differentiated areas, at least in developing CNS, as denoted by its detection in the *tbr+*like mantle surrounding the forebrain ventricle at 24 hpf. Indeed, the presence of *prr12b-*201 transcripts in the ciliary marginal zone of the 72-hpf eye represents the unique and incontrovertible sign of its association with a proliferative niche. 

However, the analysis of the *prr12* spatial distribution offers the chance for several additional observations. First of all, *prr12* regionalization in embryos and larvae corroborates data on *PRR12* enrichment in the embryonic brain. Indeed, *prr12a-*201 is found in the cerebellum primordium at 24 hpf. Moreover, both isoforms are expressed in the adult cerebellum and in the dorsal pallium, which is considered homologous to the human brain cortex [51], reinforcing the continuity with known data on *PRR12* tissue distribution in mammals. Yet, most importantly, *prr12* transcripts are found in a plethora of areas that justify *PRR12* variants as being causative of multi-system abnormalities. Indeed, syndromic *PRR12*-derived features comprise behavioral alterations such as ADHD and ASD [7,8], two NDDs characterized by impairments affecting the social sphere [52,53]. Noteworthy, our results showed that *prr12* isoforms are found in the lateral (Dl) and medial (Dm) divisions of dorsal telencephalon, Vv, the dorsal nucleus of dorsal telencephalon (Vd), ATN, cerebellum, which are considered part of the subcortical social brain [54]. Moreover, both NDDs imply an imbalance in neurotransmission involving dopamine (DA) and GABA [55,56]. Embryonic *prr12* expression may disclose a certain degree of correlation with both the DAergic and GABAergic ganglia. In fact, the telencephalic and diencephalic *prr12b*-positive territories in 24-hpf embryos seem to anatomically lie in brain areas hosting *tbr2*+ and *otpb*+ DAergic precursors, homologs to mammalian basal ganglia, as well as *dlx2a*-positive cells, which give rise to the diencephalic GABAergic system [51,57,58]. Furthermore, Otpb/TH double-stained cells in the TPp of the adult brain are considered homologous to mammalian A8-10 dopaminergic groups. This suggests that they could be an embryonic derivative of basal progenitors in otpb+ territories identified at 24 hpf, thus implying a correlation with the ventral tegmental area/substantia nigra (VTA/SN) of higher vertebrates [31,59]. 

Interestingly, DAergic imbalance in basal ganglia reward circuitry is considered causative of some of the ASD and ADHD-associated symptoms [60,61]. In teleost fishes, reward-inducing stimuli activate the Dm and the ENT [35,62], two areas that we have found to be *prr12*-positive.

Expression of *dlx2a* in the hindbrain promotes the survival of neural crest-derived sensory and cartilage components [63]. Enrichment of *prr12a* and *prr12b* in the same territories may account for the patient’s brachy- and microcephaly [7]. Additionally, there is a close association between *prr12* and the olfactory system. Odor disfunction has been recently implied in ASD/ADHD through a mechanism involving DAergic transmission [64,65,66]. Anyway, both ASD and ADHD are closely related to emotional processing [52,67,68]. Although such circuits are still not completely characterized in zebrafish, *prr12* mRNAs are found in several nuclei that are variably involved in this behavioral output. For instance, the ventral part of the ventral telencephalon (Vv) is homologous to the septum in higher vertebrates, and in mammals, there is a limbic projection from the septum to the medial habenula that is probably part of anxiety and fear pathways [51,69,70,71]. Thus, the zebrafish Vv habenular projection could represent a limbic septo-habenular pathway. Furthermore, the zebrafish thalamus, comprising the proper thalamus and habenular nuclei, homologous to mammals habenula, processes emotional information [38,71]. Interestingly, habenula involvement in ASD is largely known [61].

Non-syndromic *PRR12* variants are mainly characterized by eye defects [9]. As suggested by our histological results, both *PRR12* zebrafish orthologs are variably associated with retinal development and the visual system. In particular, the *prr12b*+ anterior-most cluster in 24-hpf embryos seems to recapitulate the *six6a* expression pattern at 24 hpf [72]. Notably, this gene is involved in neural retinal development and is an acknowledged ortholog of the mammalian SIX6 gene, whose mutations are reported to cause iris coloboma [9,72]. The coexpression of these two genes within the same regions supports the hypothesis that PRR12 might impact both eye and brain development. This influence could occur through PRR12’s ability to interact with both DNA and proteins that play different roles during the early phases of central nervous and visual system formation, as suggested by Chowdhury et al. [1].

In conclusion, the conserved gene sequence and structure of the human *PRR12* gene and its zebrafish orthologues, along with the expression of *prr12a* and *prr12b* in brain regions corresponding to those affected by human *PRR12* deficiencies, suggest that zebrafish could be a valuable model organism for studying *PRR12*-related neurodevelopmental disorders.

## Data Availability

The original contributions presented in the study are included in the article/Supplementary Material, further inquiries can be directed to the corresponding author.

## Supplementary Materials

The following supporting information can be downloaded at: https://www.mdpi.com/article/10.3390/genes15040492/s1, Figure S1: Structure comparison of human *PRR12* and zebrafish *prr12* genes; Figure S2: Evaluation of PRR12 protein domains conservation in zebrafish; Figure S3: Evaluation of PRR12 protein domains; Figure S4: RNA-Seq profile of *prr12* isoforms; Figure S5: Expression pattern of *prr12a*-202; Figure S6: Evaluation of *prr12* sense control probes.
