# Supplementary material for: Shedding a Light on Dark Genes: A Comparative Expression Study of PRR12 Orthologues during Zebrafish Development
Source: Genes (Basel). 2024 Apr 15;15(4):492. doi: 10.3390/genes15040492 (PMC11050278; doi:10.3390/genes15040492)
Supplement: Supplementary file 1 [file genes-15-00492-s001.zip › Supplementary Figures Legends.pdf]

Supplementary Figure S1 Structure comparison of human *PRR12* and zebrafish *prr12* genes. Exon-intron distribution of human *PRR12* and zebrafish *prr12a* and *prr12b* isoforms. The black dashed box outlines transcript alignment of the high conserved initial part of exon 4 of the *PRR12*, *prr12a* and *prr12b* main isoforms. Asterisks (*) indicate identical nucleotides. Identity percentage for exon 4 transcripts among human and zebrafish 201 isoforms are the following: 59.55% *PRR12 vs. prr12a*; 60,14% *PRR12 vs. prr12b*. Abbreviations: ***Hsa***: *Homo sapiens;* **Danre**: *Danio rerio*

Supplementary Figure S2 Evaluation of PRR12 protein domains. A-E Multiple Clustal alignment of PRR12-201, Prr12a-201, Prr12b-201 Glycine-rich 335-685aa (A), Proline-rich 764-1545aa (B), Proline-rich 1666-1737 (C) domains. Tables in A-C represent percentage identity matrices for each set of sequence alignment. Red ovals in D and E highlight the presence/absence of specific lysine (D) and serine (E) aminoacidic residues among human and zebrafish proteins. Abbreviations: ***Hsa***: *Homo sapiens;* **Danre**: *Danio rerio*

Supplementary Figure S3 *PRR12* and *prr12* are associated to the same paralog gene. Modified version of Ensembl phylogenetic tree showing zebrafish evolutionary maintenance among mammals (green background) and bony fishes (pink background) of *qser1* (in red), ortholog of the human *QSER1* (in red in the Primates node, highlighted by the purple background), as paralog of *prr12* genes (in blue). All the reported genes derive from a common ancestor in Jawed vertebrates.

Supplementary Figure S4 RNA-Seq profile of *prr12* isoforms. Publicly available *prr12a-201* (A) and *prr12b-201* (B) expression data at increasing developmental stages. Abbreviations: **TPM**: transcripts per million; **hpf**: hours post fertilization; **dpf**: days post fertilization

Supplementary Figure S5 Expression pattern of *prr12a*-202 A-B Relative expression analysis of *prr12a*-202 assessed by real time-PCR in embryos (A) and adult specimens (B). Ct values for each timepoint are normalized to *rps11* gene. In (A), asterisks (*) indicate statistical comparisons between each stage and dome stage; hash marks (#) indicate statistical comparisons between each stage and 50% epiboly stage; degree symbol (°) indicate statistical comparison between each stage and bud stage. Data are expressed as mean ± SD. ANOVA followed by Tukey post-hoc test: *$p<0.05$, **$p<0.01$; #$p<0.05$; °$p<0.05$, °°$p<0 .01$. N=3 independent experiments. In (B), Unpaired t test with Welch's correction: *$p<0.05$. N=3 independent experiments

Supplementary Figure S6 Evaluation of *prr12* sense control probes. Whole-mount in situ hybridization using *prr12a*-201 (A) and *prr12b*-201 sense control probes to assess anti-sense signal specificity in the several developmental stages analysed. Scale bars: 250 µm
