# Supplementary figures and images for: Shedding a Light on Dark Genes: A Comparative Expression Study of PRR12 Orthologues during Zebrafish Development
Source: Genes (Basel). 2024 Apr 15;15(4):492. doi: 10.3390/genes15040492 (PMC11050278; doi:10.3390/genes15040492)

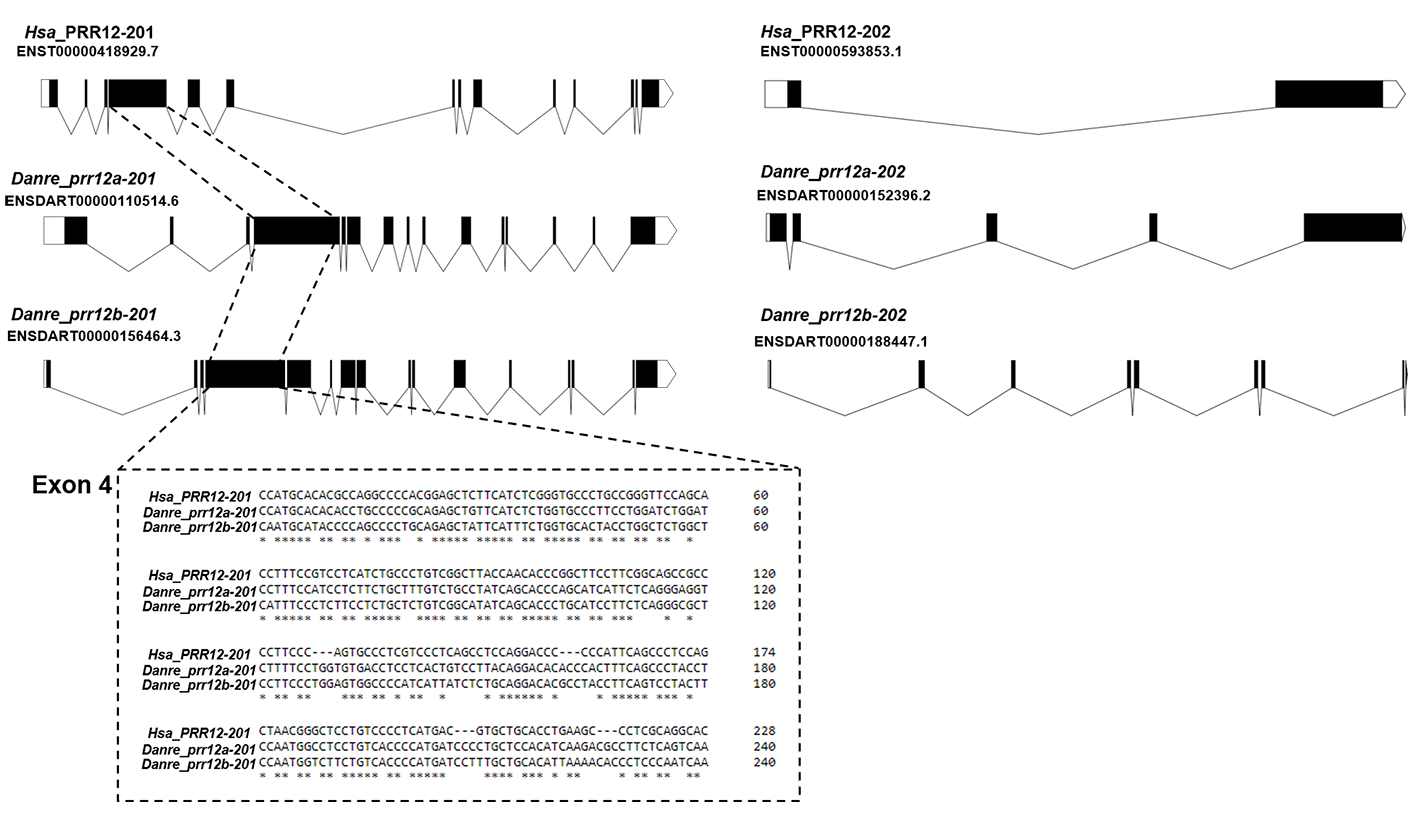

Supplement: Supplementary file 1 [file genes-15-00492-s001.zip › Supplementary Figure S1.tif]

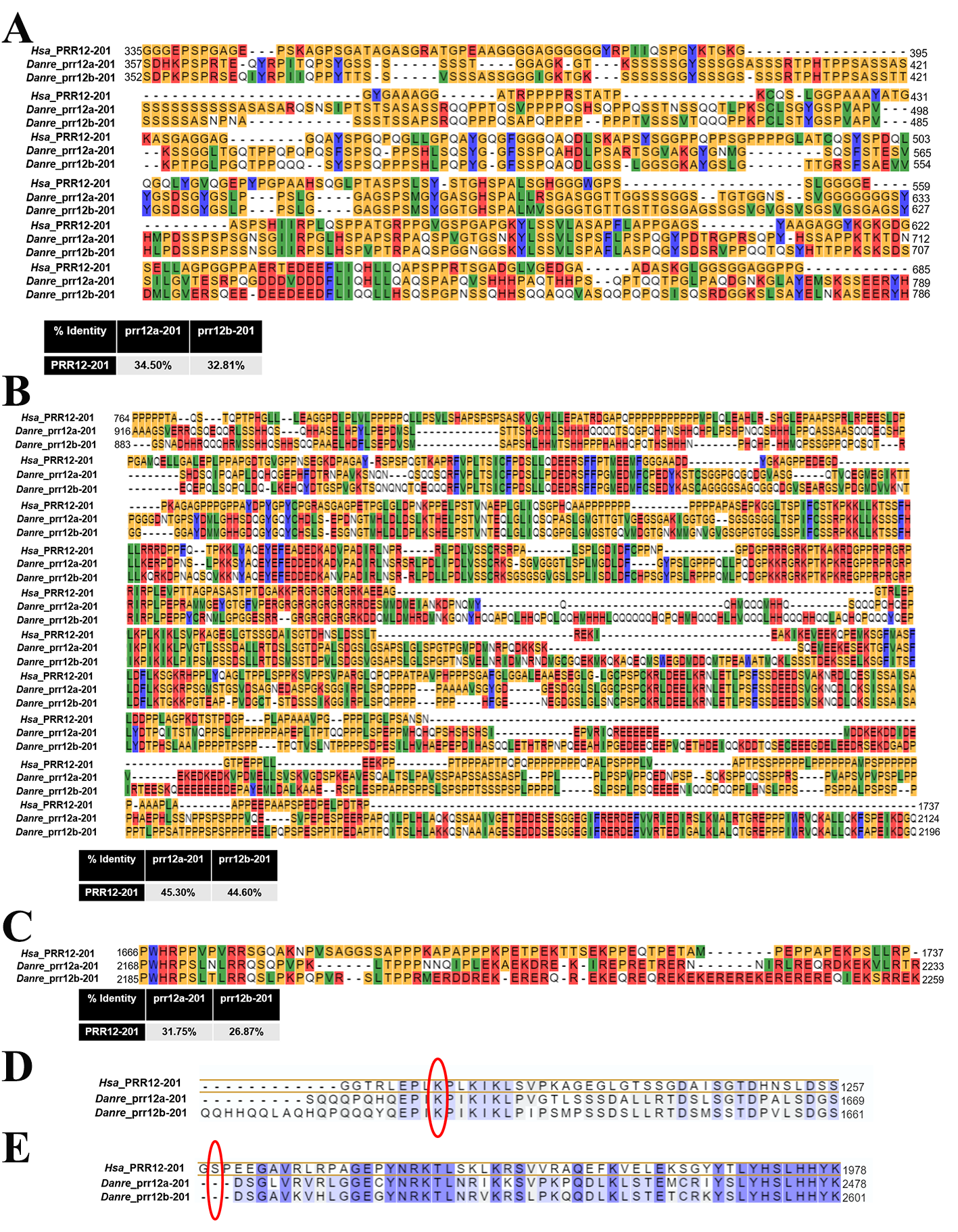

Supplement: Supplementary file 1 [file genes-15-00492-s001.zip › Supplementary Figure S2.tif]

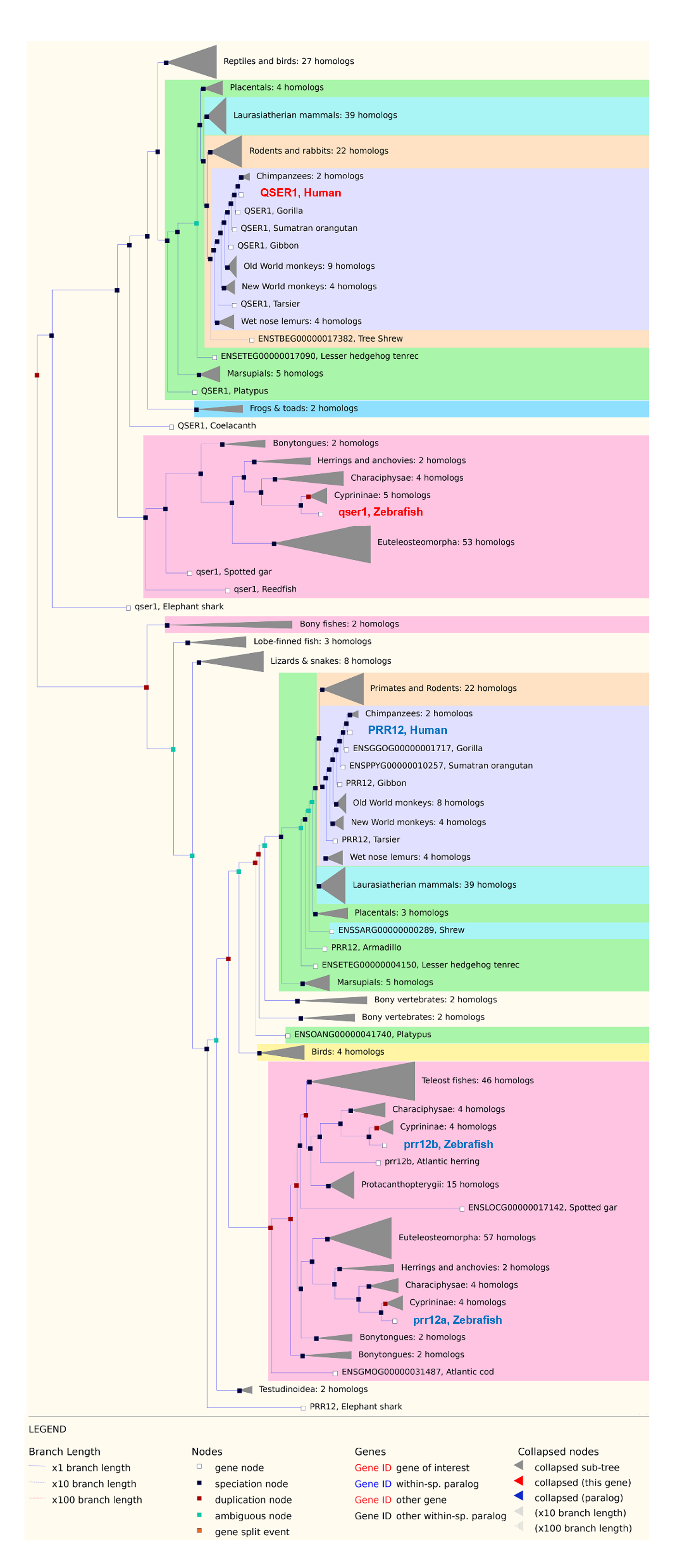

Supplement: Supplementary file 1 [file genes-15-00492-s001.zip › Supplementary Figure S3.tif]

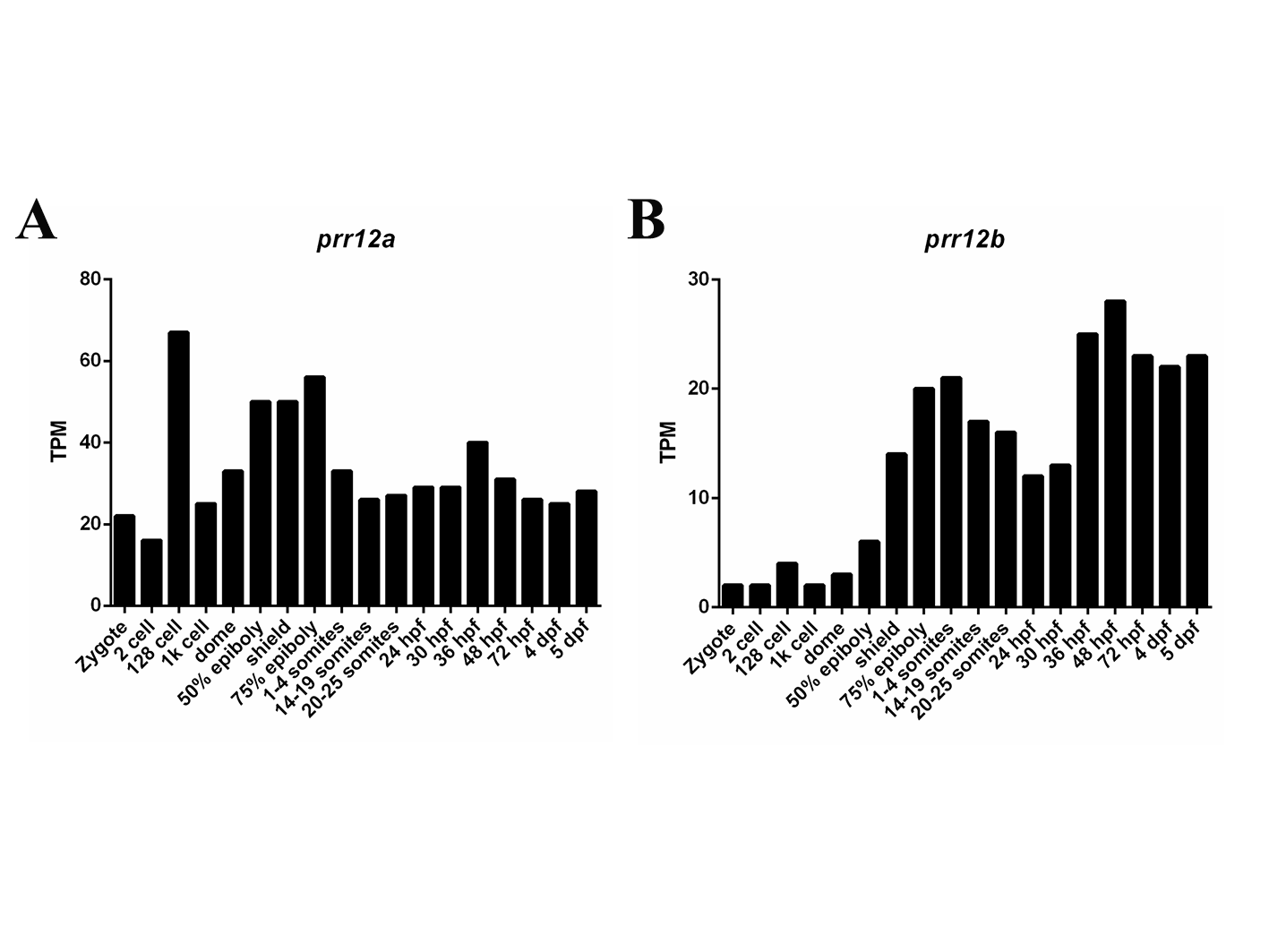

Supplement: Supplementary file 1 [file genes-15-00492-s001.zip › Supplementary Figure S4.tif]

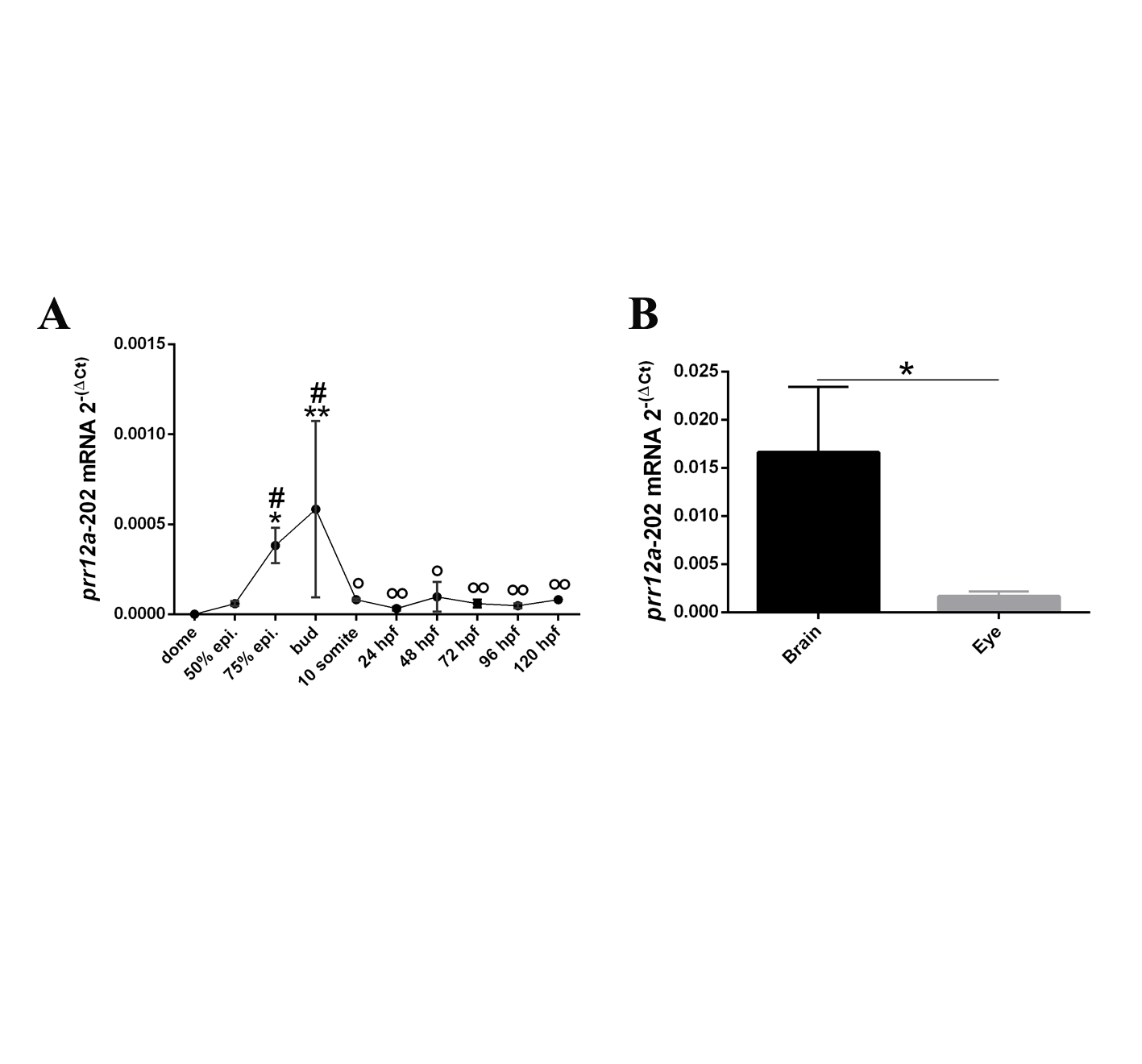

Supplement: Supplementary file 1 [file genes-15-00492-s001.zip › Supplementary Figure S5.tif]

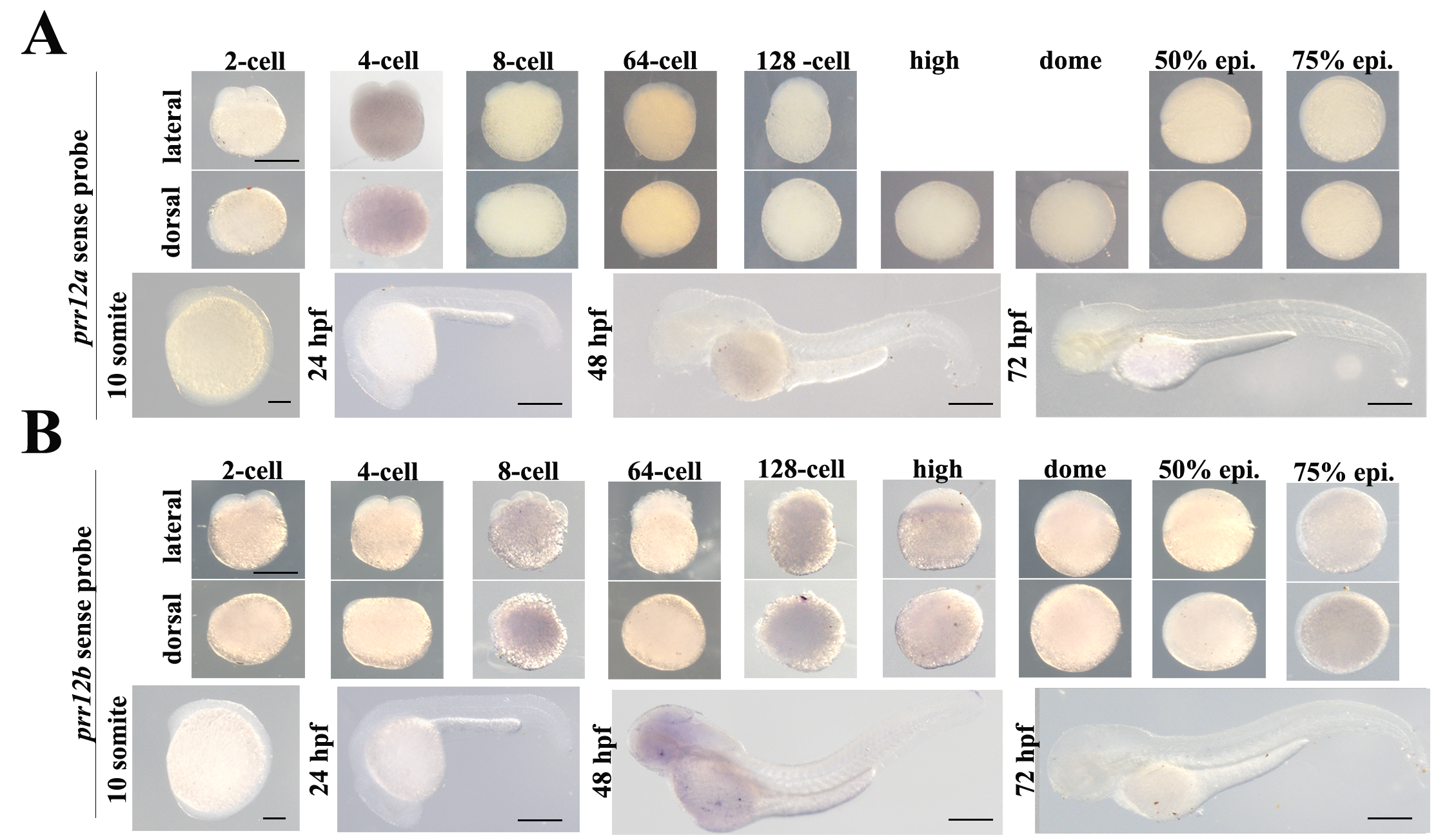

Supplement: Supplementary file 1 [file genes-15-00492-s001.zip › Supplementary Figure S6.tif]
